# A Case of Alarming Hemorrhage from the Bowels in a Little Girl, Caused by the Detachment of a Polypus

**Published:** 1846-05

**Authors:** M. H. Fee

**Affiliations:** Leatherwood, Indiana


**Art. IV.**—*A Case of Alarming Hemorrhage from the Bowels in a little girl, caused by the detachment of a Polypus.* By Dr. M. H. Fee, of Leatherwood, Indiana.

On the 13th of last September, I was hastily summoned to visit C. M., daughter of C. A. B., æt. 10 years. On my arrival I learned of Mrs. B. that an hour before, the little girl had a bloody discharge from her bowels amounting, according to her estimate, to something more than a quart. There was also discharged at the same time, what I considered, from description, to be a polypus, said by the parents to be somewhat larger than a partridge egg, of oval form, studded over the outer surface with hemispherical prominences, filled with a sero-purulent fluid, the peduncle being about the size of a large goose quill. It was left on the ground where it was examined, and before my visit had been swallowed by the poultry. I found the little patient much prostrated, with pulse scarcely perceptible, cold extremities, a sense of stricture at the præcordia, and distressing nausea. I directed warmth to be applied to the feet, while I prepared and administered the following mixture: tinct. opii gtt. 20, spts. ammon. gtt. 10, acet. plumb. gr. 1½.

In a short time after the administration of the draught, there was decided relief of the præcordial distress, as well as of the nausea, and the coldness of the extremities; and in about half an hour she had another bloody discharge, entirely different from the former; this being mostly clots of grumous blood while in the former, the blood was perfectly liquid. The last discharge amounted to about three gills. In an hour and a half after the administration of the draught, I repeated the remedy in half the dose.

Returning a few hours afterwards, I found my patient suffering with acute pain in the head which was aggravated by the least jar or noise, her face flushed, and eyes injected. At first sight I feared lest I had carried stimulation too far; after feeling the pulse, I became satisfied that this was not the case, but that the symptoms arose from loss of blood. In accordance with this view of the case I mixed twenty drops of the spirits of ammonia, with four tablespoonfuls of water, and gave a tablespoonful of the mixture, every fifteen minutes until three doses were given; when her head became easy, and she fell into a gentle slumber. I left some laudanum and acetate of lead to be given in case the hemorrhage should return.

Sept. 14th. Found my patient laboring under some excitement, and headache, from a chill which she had at nine o'clock this morning. The fever was soon reduced by spts. nitre; and her head relieved by cold applications. Ordered castor oil ℥ss., oil of turpentine 15gtt., to be taken immediately. Had had one small grumous discharge during the night.—Discontinued the saturnine preparation, and the opiates.

Sept. 15th. Found my patient in a strong rigor. It was now clear that this was a regular intermittent of the quotidian type. Ordered a solution of 16grs. of quinine in two ounces of water, of which two teaspoonfuls to be taken every two hours, after the decline of the slight febrile excitement; also castor oil ℥ss., and oil of turpentine 20gtt., to be taken this afternoon. The oil and turpentine of yesterday had produced two alvine dejections, feculent, and free from every trace of blood.

16th. Found my patient greatly improved; no chill; two feculent discharges; had been up, and walked from the bed to the fire-place, without assistance. Ordered quinine to be continued in small doses, hourly, for a few days, with wine, and generous diet; bowels to be moved by castor oil and spirits turpentine in the afternoon.

17th. This morning made my last visit to my patient; found her sitting up; fast improving in appearance and strength. From this time, her convalescence was rapid.

This little girl had been, for several years, of a sickly appearance, sallow complexion, with constant præcordial distress, and weak digestive powers. She was thought to be troubled with worms.

I had been frequently called to see her, and had invariably prescribed for the verminose symptoms, but without at any time effecting any considerable discharge of worms, the symptoms persisting, though with abated severity. She even became paralytic at times; and frequently quite unconscious, as if sitting up asleep, and in that posture showed no tendency to fall. In a word, the case exhibited, at times, all the characteristic marks of catalepsy. Suspecting that tapeworm might be the cause of the evil, I had taken steps to procure remedies for it when the discharge of blood, and of the polypus came on, and changed my diagnosis of the affection. It may still be a question whether this polypus, so inconsiderable in size, was capable of producing so much constitutional disturbance, and especially so serious a derangement of the nervous system. I am free however to say, that notwithstanding the apparent inadequacy of the cause, it was, to my mind, the sole cause of the complaint. In proof of the correctness of this opinion we have the fact, that the girl has regained her health perfectly since the polypus passed away, and is now free from all symptoms of disease.

February 24th, 1846.

